# Comparison of nanovesicles derived from *Panax notoginseng* at different size: physical properties, composition, and bioactivity

**DOI:** 10.3389/fphar.2024.1423115

**Published:** 2024-07-22

**Authors:** Xiaohang Chen, Liyu Huang, Mengyuan Zhang, Shuoqi Lin, Jing Xie, Hengyi Li, Xing Wang, Youguang Lu, Dali Zheng

**Affiliations:** ^1^ Fujian Key Laboratory of Oral Diseases, School and Hospital of Stomatology, Fujian Medical University, Fuzhou, China; ^2^ Department of Preventive Dentistry, School and Hospital of Stomatology, Fujian Medical University, Fuzhou, China; ^3^ Department of Human Anatomy and Histology, and Embryology, School of Basic Medical Sciences, Fujian Medical University, Fuzhou, China; ^4^ Shanxi Medical University School and Hospital of Stomatology, Taiyuan, China

**Keywords:** plant derived nanovesicles, *Panax notoginseng*, squamous cell carcinoma, tuber size, composition, bioactivity

## Abstract

**Aim:**

Plant-derived nanovesicles have emerged as potential agents for combating tumors. In this study, we investigated the inhibitory effects of *Panax notoginseng*-derived nanovesicles (PnNVs) on the proliferation and migration of squamous cell carcinoma. Additionally, we explored the relationship between plant tuber size and the physical properties, composition and bioactivity of these nanovesicles.

**Methods:**

We isolated PnNVs from *Panax notoginseng* tubers of varying sizes: small-sized (s_PnNVs), medium-sized (m_PnNVs) and large-sized (l_PnNVs), and evaluated for size, potential, and morphology. Cellular uptake efficiency was assessed using confocal microscopy and flow cytometry. The ability of different PnNVs to inhibit oral squamous cell carcinoma cells was evaluated using plate cloning, CCK8 assay, and scratch healing assay. Off-target metabolomics was used to compare metabolic compounds of different PnNVs.

**Results:**

Our findings revealed that s_PnNVs exhibited lower potential but had the highest cellular uptake efficiency, whereas m_PnNVs were characterized by the smallest size and lowest cellular uptake efficiency. Notably, m_PnNVs demonstrated the most effective inhibition of squamous cell carcinoma growth and migration. Compositional analyses showed that PnNVs were rich in proteins and contained lower levels of RNA, with l_PnNVs having the highest protein content. Furthermore, untargeted metabolomics analysis revealed a significant increase in the expression of specific antitumour-related metabolites in m_PnNVs compared to s_PnNVs and l_PnNVs.

**Conclusion:**

Overall, our results underscore the influence of plant tuber size on the bioactivity of the nanovesicles from which they are derived, emphasizing its importance for experimental design and study reproducibility.

## Highlights

1. *Panax notoginseng* derived nanovesicles (PnNVs) inhibit squamous cell carcinoma proliferation and migration, with varying potency from different size.
2. PnNVs from *Panax notoginseng* at different size differ in morphology, potential distribution, particle size distribution, and composition.
3. PnNVs from *Panax notoginseng* at different size show different cellular endocytosis efficiency.

## 1 Introduction

Plant-derived nanovesicles (PDNVs) are lipid bilayer vesicles derived from plants, comprising plant-derived lipids, proteins, RNA, and metabolic compounds (Chen et al., 2023a). Serving as a natural drug encapsulation system, PDNVs possess the capability to improve drug bioavailability and exhibit enhanced cellular uptake owing to their lipid bilayer structure (Niu et al., 2021; Xiao et al., 2022). Furthermore, PDNVs are highly adaptable and can function as targeted drug delivery vehicles, showcasing considerable promise for utilization in fields such as antitumor treatment and tissue regeneration (Chen et al., 2023a; Chen et al., 2023b). Numerous studies have highlighted the potential of PDNVs in the realm of anti-tumor research (Ly et al., 2023), with examples including bitter melon (Yang et al., 2021), cucumber (Chen et al., 2022), and artemisia annua (Liu et al., 2023). The exploration for PDNVs exhibiting anti-tumor properties holds significant promise.

*Panax notoginseng*, a plant known for its diverse array of over 200 compounds with demonstrated antitumor (Zhang et al., 2022), anti-inflammatory (Zheng et al., 2022), haemostatic (Liu et al., 2014), and antioxidant properties (Xu et al., 2023a), is commonly utilized in herbal formulations like Yunnan Baiyao (Zhu et al., 2017). Of note, the tuber of *Panax notoginseng* plays a crucial role in contributing to its medicinal efficacy (Zheng et al., 2022). Previous studies have isolated nanovesicles from *Panax notoginseng* tuber (PnNVs) and demonstrated their ability to mitigate ischemia-reperfusion injury by modulating microglia polarization (Li et al., 2023). Furthermore, compounds from *Panax notoginseng tuber*, such as *Panax notoginseng* saponins and other components, have shown promising antitumor activity against hepatocellular carcinoma (Liu et al., 2021a), and breast cancer (Wang et al., 2014). Nevertheless, the anti-tumor efficacy of PnNVs, as potent herbal agents, remains incompletely understood. Squamous cell carcinoma is the predominant tumor pathology, prompting our initial assessment of the efficacy of PnNVs in suppressing the proliferative and migratory characteristics of squamous cell carcinoma. Our investigation revealed variations in the inhibitory effects of PnNVs based on their size, raising questions about the influence of factors such as tuber size on the bioactivity of these nanovesicles.

Numerous studies on PDNVs currently suffer from a lack of standardized criteria for plant selection, leading to potential challenges in study reproducibility. For example, certain plants such as turmeric (Gao et al., 2022) and ginseng (Kim et al., 2023) are commonly utilized for their tubers in the isolation of nanovesicles, yet the impact of varying sizes on the physical properties and bioactivities of PDNVs is frequently overlooked. It is essential to consider factors such as growth stage, and other variables, as these may significantly influence the composition and concentration of active compounds within PDNVs (Liu et al., 2020; Han et al., 2023). Hence, it is imperative for plant selection to consider these factors in order to ensure the validity of research findings.

To fill this research void, *Panax notoginseng* was chosen as the focal point of investigation. This study presents the initial comprehensive examination of nanovesicles derived from *Panax notoginseng* tubers of varying sizes, encompassing distinctions in physical attributes such as shape, dimensions, and zeta potential dispersion, variances in cellular uptake and bioactivity, and distinctions in the content composition. The primary objective of this study was to provide a theoretical basis for the efficacy of PnNVs and the potential for identifying novel therapeutic uses for PnNVs. Additionally, emphasis was placed on the careful selection of tuberous plants in subsequent experimental design to enhance the replicability of the study in future research endeavors.

## 2 Materials and methods

### 2.1 Cell culture

The SCC-7 cells, a mouse squamous cell carcinoma cell line, were obtained from Yimo Bio (Xiamen, China), while the LN4 cells were derived from the CAL-27 cell line and purified following four rounds of lymph node metastases. Our research group previously engineered this cell line to exhibit heightened lymph node metastatic properties. The cell line was confirmed through short tandem repeat (STR) analysis (Gan et al., 2018). Both cell lines were maintained in Dulbecco’s modified eagle’s medium (DMEM) (Gibco) supplemented with 10% fetal bovine serum (FBS) (PAN) at 37°C in a 5% CO_2_ incubator. Passages were conducted through trypsin digestion (Gibco) upon reaching 80% cell confluence.

### 2.2 Isolation of PnNVs

*Panax notoginseng* of varying sizes was obtained from a single farm in Wenshan, Yunnan, China. The fresh *panax notoginseng* tuber were initially washed with water and then rinsed with distilled water. Subsequently, the tubers were diced, squeezed, and filtered, to produce a supernatant. The supernatant underwent sequential centrifugation steps at 2,000 g for 10 min, 5,000 g for 20 min, and finally at 10,000 g for 30 min, with each step involving the separation of sediment and preservation of the supernatant. The supernatant was subjected to centrifugation at 100,000 g for 30 min to prevent nanovesicle aggregation (Chen et al., 2022), followed by resuspension in phosphate-buffered saline (PBS). This resuspended solution underwent two subsequent rounds of centrifugation at 100,000 g for 30 min. The resulting precipitate was resuspended in PBS solution, filtered through a 0.22 μm filter membrane, and stored at 4°C in a refrigerator.

### 2.3 Physical characterization of PnNVs

The nanovesicles were characterized morphologically using transmission electron microscopy (FEI TecnaiG2, Japan). The PnNVs were fixed with 4% paraformaldehyde by placing a copper mesh on top and inverting it for 20 min, followed by three washes with ddH_2_O. Subsequently, the samples were stained with a 2% phosphotungstic acid solution (pH = 7) for 2 min and washed three more times with ddH_2_O before being dried for further observation. Particle size and zeta potential distribution were determined using dynamic light scattering (DLS) (Anton Paar, Austria).

### 2.4 Cellular uptake of PnNVs

Cells were initially cultured in 12-well plates to allow for adherence. Subsequently, PnNVs, labeled with DIO fluorescent dye (UElandy), were added to the cells, and the uptake of PnNVs was observed via confocal microscopy (Olympus, Japan) after a 24-h incubation period. The cytoskeleton was stained with Phalloidin (UElandy), while the nucleus was stained with DAPI (Beyotime). Furthermore, cells were cultured in 6-well plates and exposed to DIO-labeled PnNVs. The efficacy of cellular uptake of PnNVs was assessed at 1, 12, and 24 h using flow cytometry (BD Accuri™ C6 plus, America).

### 2.5 Cell proliferation assay

Cells were cultured in 96-well plates, followed by replacement of the medium after 24 h. Subsequently, varying concentrations of PnNVs were added into the wells. A 10 µL CCK-8 working solution (Invitrogen) was then added, and after 1-h incubation period, optical density values were assessed at 450 nm to determine cell viability.

Cell viability was quantified using the formula:

$$\text{Cell viability}\left(\%\right)=\left({\text{optical}\;\text{density}\;\left(\text{OD}\right)}_{\text{treated}\;\text{sample}}-{\text{OD}}_{\text{blank}}\right)\;/\;\left({\text{OD}}_{\text{control}\;\text{sample}}-{\text{OD}}_{\text{blank}}\right)\times 100\%$$

### 2.6 Plate cloning experiment

Cells were initially plated in 6-well plates and incubated for 24 h before the medium was removed. Subsequently, the cells were treated with PnNVs at a concentration of 25 μg/mL. Upon the formation of clones, the cells were rinsed with PBS, fixed with 4% paraformaldehyde, stained with crystal violet (Beyotime) for 10 min, washed with running water, and finally imaged and recorded.

### 2.7 Scratch experiment

Cells were cultured in 12-well plates until reaching 95% confluence, at which point a cross-shaped scratch was created using a sterile P-200 pipette tip. Following PBS washing, PnNVs were added at concentrations of 25 μg/mL and 50 μg/mL. Images were captured before and after PnNVs addition, as well as during healing of the cell at the same position.

The wound healing rate was calculated using the formula:

$$\text{Wound healing rate}=\left(\text{initial blank area }-\text{ healed blank area}\right)/\left(\text{initial blank area}\right)\times 100\%.$$

### 2.8 Compositional identification of PnNVs

#### 2.8.1 RNA

RNA was extracted from PnNVs by lysing the PnNVs with trizol (Invitrogen), followed by extraction, precipitation, and washing steps using chloroform, isopropanol, and 75% anhydrous ethanol (prepared with DEPC water), respectively. The RNA was then quantified using a Nanodrop (Thermo, United States). Subsequently, a 1% agarose gel was prepared, and 1 μg PnNVs’ RNA was loaded onto the gel. Electrophoresis was carried out at 110 V for 30 min, and images were captured for analysis (Bioscience, China).

#### 2.8.2 Proteins

Proteins were extracted from PnNVs through a process involving lysis with 5 × SDS lysate and sonication, resulting in homogeneous samples. These samples were then subjected to electrophoresis at 150 V for 60 min using a vertical electrophoresis device. The gels were stained with Coomassie brilliant blue and decolorized over a 24-h period. Photographs were taken and documented.

Furthermore, the quantity of RNA extracted was verified by measuring the concentration of each extraction with a Nanodrop and calculating the total RNA extracted based on the volume. The concentration of each protein extraction was assessed utilizing the bicinchoninic acid (BCA) protein quantification method, and subsequently multiplied by the volume to astertain the total protein yield.

### 2.9 Non-targeted metabolomics analysis of PnNVs

The PnNVs were subjected to vortexing for a duration of 1 min, followed by the addition of an appropriate sample size to 400 µL of methanol solution. Subsequently, the resulting mixture underwent vortexing for 1 min and was then subjected to centrifugation at 12,000 rpm for 10 min at 4°C. The entirety of the supernatant was collected, concentrated, and subjected to drying. Following the drying process, the sample was reconstituted by the addition of 150 µL of a 2-chloro-L-phenylalanine solution (4 ppm) prepared in 80% methanol in water. The supernatant was subsequently filtered through a 0.22 μm membrane, and the resulting filtrate was transferred to the detection vial for liquid chromatography-mass spectrometry (LC-MS) non-targeted metabolomics analysis (Panomix Biomedical Tech Co., Ltd., Suzhou, China).

### 2.10 Statistical analyses

Numerical results were presented as mean ± standard deviation. Statistical analysis involved the use of t-tests for comparisons between two groups and one-way Analysis of Variance (ANOVA) for comparisons among three groups. Significant differences identified by ANOVA were further examined through *post hoc* tests for specific group differences.

## 3 Results

### 3.1 Differences in the physical properties of PnNVs

**FIGURE 1:**
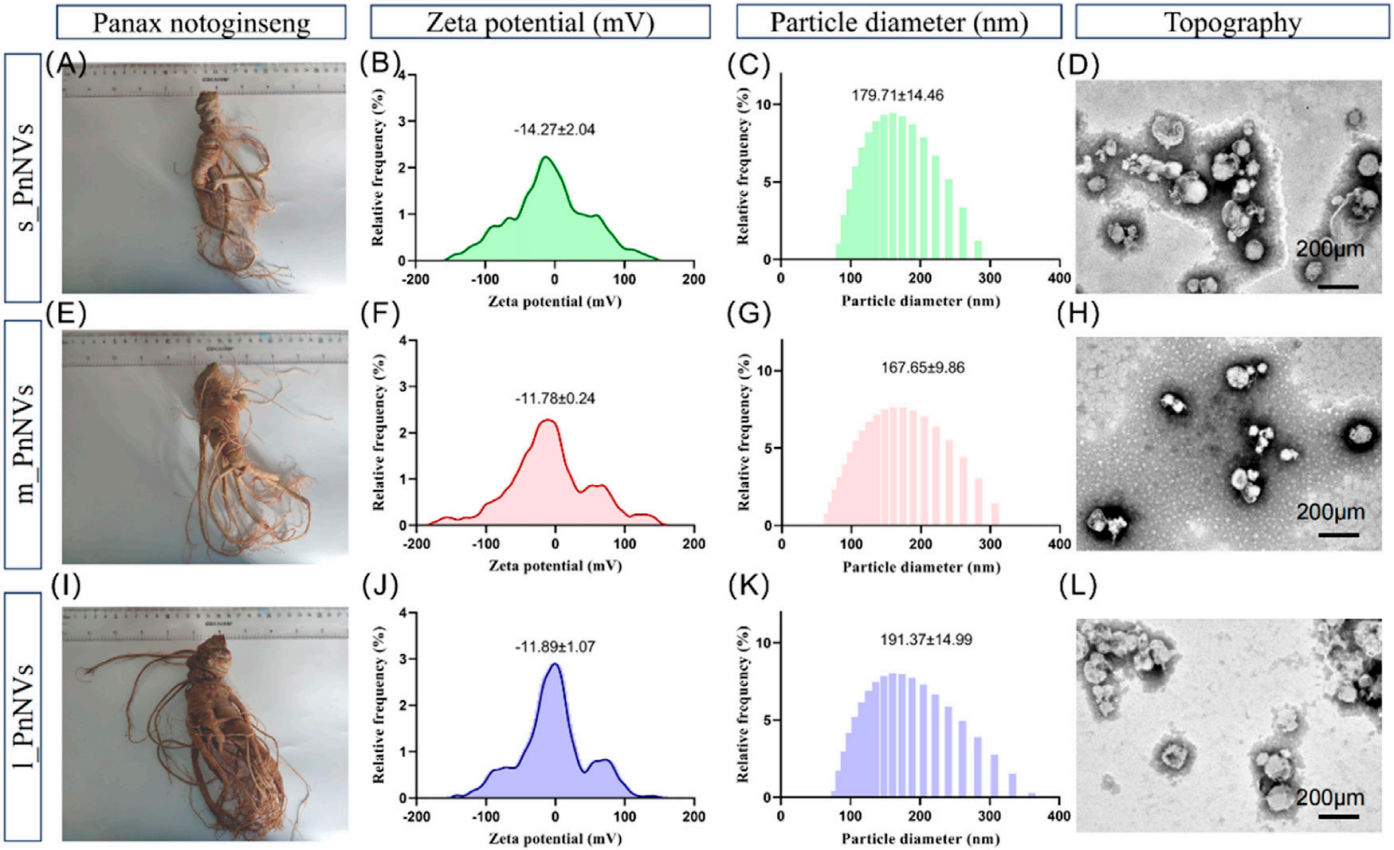
Physical properties of *Panax notoginseng*-derived nanovesicles at different growth stages. **(A)** Images of *Panax notoginseng* tubers around 35 g in size; **(B)** Zeta potential of s_PnNVs; **(C)** Particle size of s_PnNVs; **(D)** Electron microscope images of s_PnNVs; **(E)** Images of *Panax notoginseng* tubers around 65 g in size; **(F)** Zeta potential of m_PnNVs; **(G)** Particle size of m_PnNVs; **(H)** Electron microscope images of m_PnNVs; **(I)** Images of *Panax notoginseng* tubers around 125 g in size; **(J)** Zeta potential of l_PnNVs; **(K)** Particle size of l_PnNVs; **(L)** Electron microscope images of l_PnNVs.

As previously indicated, PnNVs were categorized into three groups based on the size of *Panax notoginseng* tubers. Specifically, nanovesicles obtained from *Panax notoginseng* tubers weighing approximately 35 g (Figure 1A) were designated as s_PnNVs, those from tubers weighing around 65 g (Figure 1E) were designated as m_PnNVs, and nanovesicles isolated from tubers weighing approximately 125 g (Figure 1I) were designated as l -PnNVs.

The physical characteristics of nanovesicles, including particle size distribution, potential distribution, and morphology, play a significant role in determining their cellular uptake efficiency and subsequent biological activity (Behzadi et al., 2017). In this study, we conducted an evaluation of the fundamental physical properties of PnNVs. The zeta potential of s_PnNVs was measured at −11.78 ± 0.24 mV (Figure 1B), with a particle size of 179.71 ± 14.46 nm (Figure 1C). m_PnNVs exhibited a zeta potential of −14.27 ± 2.04 mV (Figure 1F), with a particle size of 167.65 ± 9.86 nm (Figure 1G). l_PnNVs had a zeta potential of −11.89 ± 1.07 mV (Figure 1J), and a particle size of 191.37 ± 14.99 nm (Figure 1K). Through the utilization of transmission electron microscopy, it was observed that all nanovesicles exhibited vesicle-like structures (Figures 1D, H, L). From these findings, it can be inferred that s_PnNVs possess the least potential, m_PnNVs have the smallest size, and both m_PnNVs and l_PnNVs demonstrate comparable potentials.

### 3.2 s_PnNVs exhibit the highest cellular uptake efficiency

**FIGURE 2:**
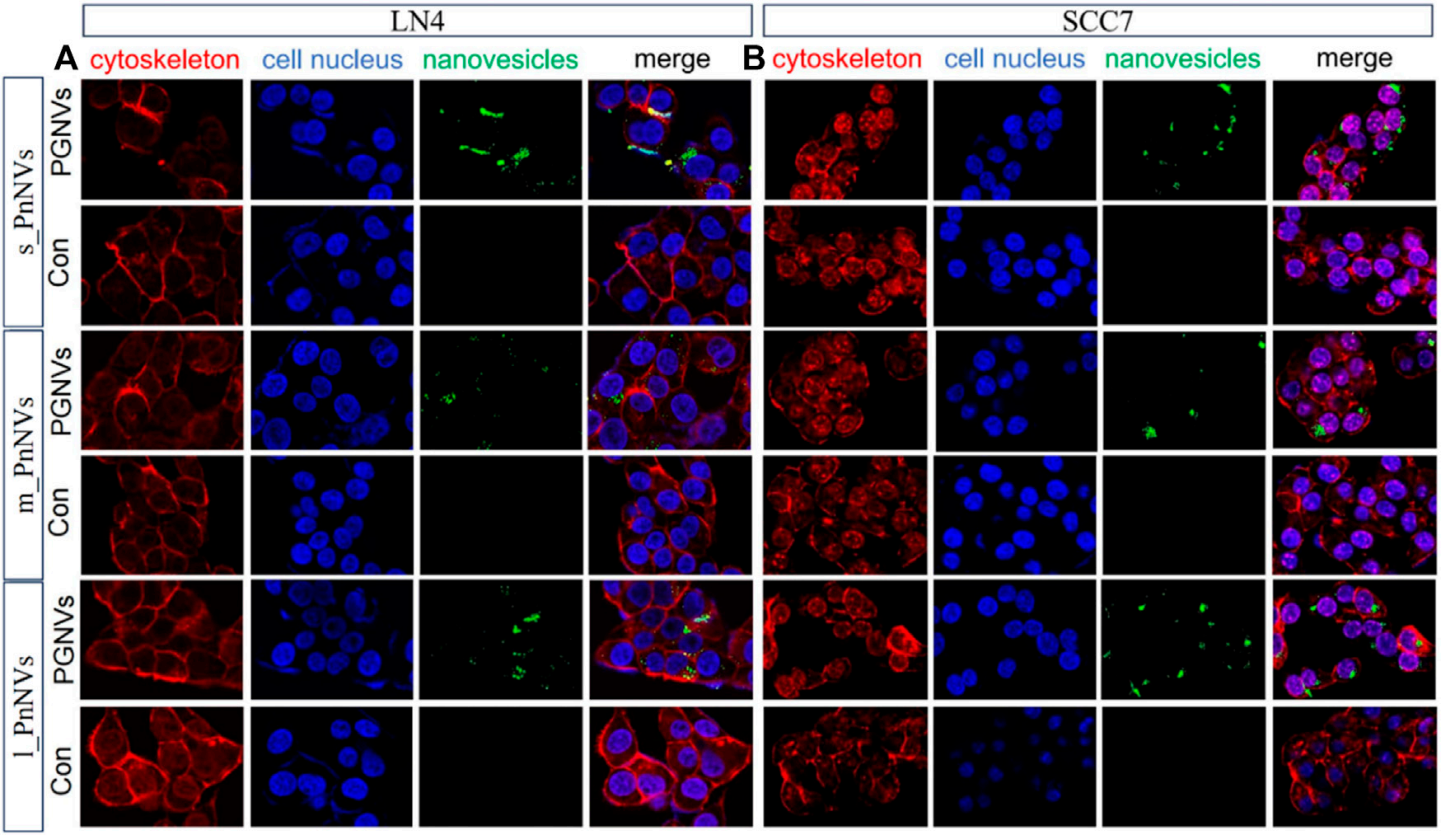
Representative images of cellular uptake of PnNVs. **(A)** Efficiency of PnNVs uptake by LN4 cells. **(B)** Efficiency of PnNVs uptake by SCC7 cells.

The cellular internalization of nanovesicles serve as the foundation for their biological impacts. In order to evaluate variances in theintracellular uptake efficacy of PnNVs, staining and co-localization analyses were conducted. Upon co-incubating DIO dye-labeled PnNVs with LN4 and SCC7 cells for 24 h, it was observed that the green fluorescent signals were notably concentrated within the cells, predominantly localized in the cytoplasm, with the most intense signals observed in the s_PnNVs group (Figure 2).

**FIGURE 3:**
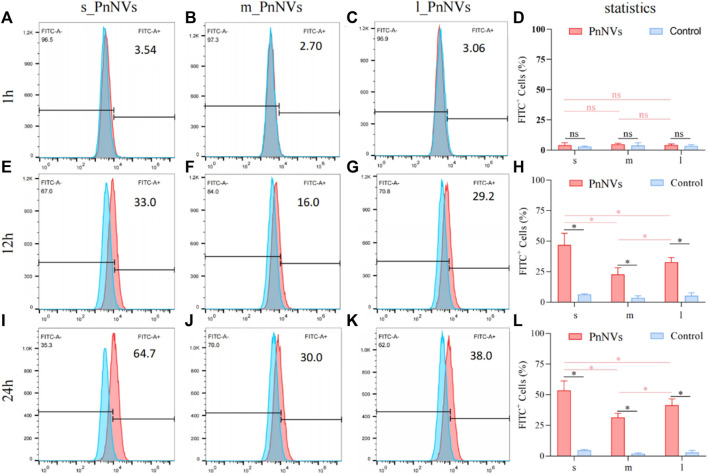
Efficiency of PnNVs uptake by LN4 cells. **(A)** Efficiency of s_PnNVs uptake by LN4 cells at 1 h. **(B)** Efficiency of m_PnNVs uptake by LN4 cells at 1 h. **(C)** Efficiency of l_PnNVs uptake by LN4 cells at 1 h. **(D)** The statistics of efficiency of PnNVs uptake by LN4 cells at 1 h. **(E)** Efficiency of s_PnNVs uptake by LN4 cells at 12 h. **(F)** Efficiency of m_PnNVs uptake by LN4 cells at 12 h. **(G)** Efficiency of l_PnNVs uptake by LN4 cells at 12 h. **(H)** The statistics of efficiency of PnNVs uptake by LN4 cells at 12 h. **(I)** Efficiency of s_PnNVs uptake by LN4 cells at 24 h. **(J)** Efficiency of m_PnNVs uptake by LN4 cells at 24 h. **(K)** Efficiency of l_PnNVs uptake by LN4 cells at 24 h. **(L)** The statistics of efficiency of PnNVs uptake by LN4 cells at 24 h.

The investigation into the efficiency of PnNVs uptake by LN4 cells was conduucted using flow cytometry. Results indicated that after 1 h, the fluorescent signals in the cells were minimal and did not exhibit a significant difference (Figures 3A–D). However, following 12 h of co-incubation, there was a notable increase in fluorescent signals within the LN4 cells (Figures 3E–H), with the s_PnNVs group showing the highest signal intensity, comprising approximately 33.0% of the total signals (Figure 3E). At the 24-h time point, the fluorescence signal of the s_PnNVs group remained the most intense, representing approximately 64.7% of the total signals (Figure 3I). Conversely, the signals emitted by m_PnNVs consistently exhibited lower intensity, accounting for only around 30% of the overall signals at the 24-h time point (Figure 3J). In contrast, l_PnNVs demonstrated an intermediate uptake efficiency (Figures 3K, L).

**FIGURE 4:**
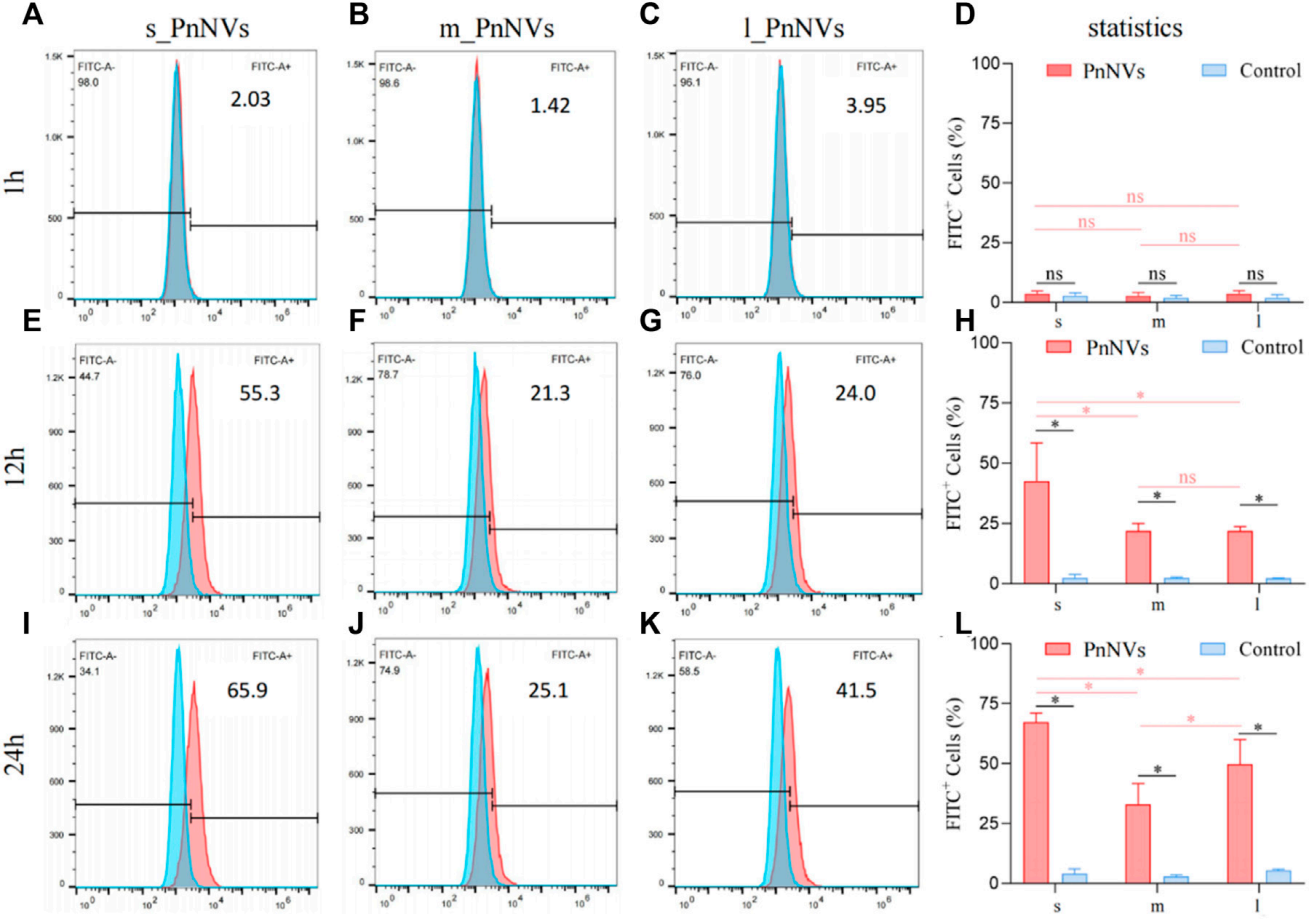
Efficiency of PnNVs uptake by SCC7 cells. **(A)** Efficiency of s_PnNVs uptake by SCC7 cells at 1 h. **(B)** Efficiency of m_PnNVs uptake by SCC7 cells at 1 h. **(C)** Efficiency of l_PnNVs uptake by SCC7 cells at 1 h. **(D)** The statistics of efficiency of PnNVs uptake by SCC7 cells at 1 h. **(E)** Efficiency of s_PnNVs uptake by SCC7 cells at 12 h. **(F)** Efficiency of m_PnNVs uptake by SCC7 cells at 12 h. **(G)** Efficiency of l_PnNVs uptake by SCC7 cells at 12 h. **(H)** The statistics of efficiency of PnNVs uptake by SCC7 cells at 12 h. **(I)** Efficiency of s_PnNVs uptake by SCC7 cells at 24 h. **(J)** Efficiency of m_PnNVs uptake by SCC7 cells at 24 h. **(K)** Efficiency of l_PnNVs uptake by SCC7 cells at 24 h. **(L)** The statistics of efficiency of PnNVs uptake by SCC7 cells at 24 h.

Furthermore, the uptake efficiency of PnNVs by SCC7 cells was evaluated. The results indicated that at 1 h, the fluorescent signals within the cells were also faint, awith no significant deviation observed compared to the control (Figures 4A–D). Following a 12-h of co-incubation period, the fluorescence signals within SCC7 cells exhibited a significant increase (Figures 4E–H). The s_PnNVs group demonstrated the highest signal intensity compared to the m_PnNVs and l_PnNVs groups, with an enhancement of 55.3% (Figure 4E). Even at the 24-h time point, the s_PnNVs group maintained the strongest fluorescence signal at approximately 65.9% (Figures 4I–K). Conversely, the m_PnNVs group were consistently displayed weaker signals, representing only about 25.1% of the total signal at the 24-h time point (Figure 4G). In contrast, the uptake efficiency of l_PnNVs fell between the other groups at 41.5% (Figures 4K, L).

The aforementioned findings indicate that the uptake efficiency of PnNVs exhibits variability across different cell types. Specifically, s_PnNVs derived from *Panax notoginseng* at a smaller size demonstrated the highest intracellular uptake efficiency, while m_PnNVs obtained from *Panax notoginseng* at a medium size exhibited the lowest intracellular uptake efficiency.

### 3.3 PnNVs were all effective in inhibiting tumor cell proliferation, with m_PnNVs being the better effectiveness

**FIGURE 5:**
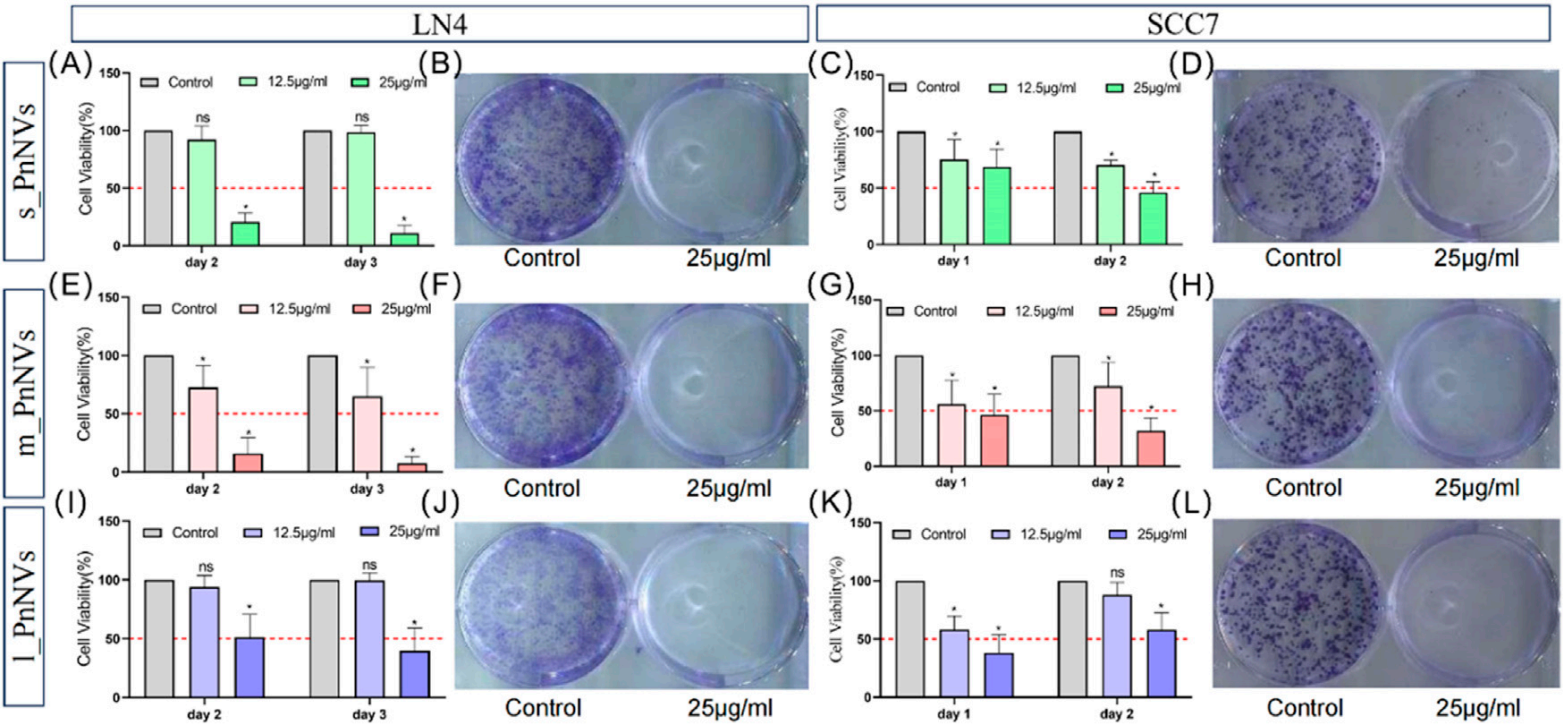
Effectiveness of PnNVs in inhibiting cell proliferative activity. **(A)** Statistical graph of inhibition of LN4 cell viability by different concentrations of s_PnNVs. **(B)** Clone formation of LN4 cells after intervention with 25 μg/mL s_PnNVs. **(C)** Statistical graph of the inhibition of SCC7 cell viability by different concentrations of s_PnNVs. **(D)** Clone formation of SCC7 cells after intervention with 25 μg/mL s_PnNVs. **(E)** Statistical graph of inhibition of LN4 cell viability by different concentrations of m_PnNVs. **(F)** Clone formation of LN4 cells after intervention with 25 μg/mL m_PnNVs. **(G)** Statistical graph of inhibition of SCC7 cell viability by different concentrations of m_PnNVs. **(H)** Clone formation of SCC7 cells after intervention with 25 μg/mL m_PnNVs. **(I)** Statistical graph of inhibition of LN4 cell viability by different concentrations of l_PnNVs. **(J)** Clone formation of LN4 cells after intervention with 25 μg/mL l_PnNVs. **(K)** Statistical graph of inhibition of SCC7 cell viability by different concentrations of l_PnNVs. **(L)** Clone formation of SCC7 cells after intervention with 25 μg/mL l_PnNVs.

Malignant proliferation is a prominent characteristic of tumors (Hanahan and Weinberg, 2000). Subsequently, the impact of PnNVs on squamous cells proliferation was investigated. Through CCK-8 assay analysis of cell viability, it was observed that m_PnNVs displayed greater efficacy in inhibiting the viability of LN4 cells and SCC7 cells (Figure 5). Specifically, at 24 h and 48 h, 12.5 μg/mL of m_PnNVs effectively inhibited LN4 cell viability, while s_PnNVs and l_PnNVs did not demonstrate inhibitory effects (Figures 5A, E, I). However, at a concentration of 25 μg/mL, all groups demonstrate effective inhibition cell proliferation viability when exposed to nanovesicles (Figures 5A, E, I).

Subsequent validation experiments conducted with SCC7 cells showed that all groups, except for l_PnNVs at 12.5 μg/mL, effectively inhibited cell proliferation viability, as evidenced by the lack of inhibitory effect on cell proliferation viability on the second day (Figures 5C, G, K). Plate cloning experiments, further revealed that 25 μg/mL of PnNVs successfully inhibited the proliferation of LN4 cells and SCC7 cells, ultimately leading to cell death (Figures 5B, D, F, H, J, L).

### 3.4 PnNVs were all effective in inhibiting tumour cell migration, with m_PnNVs being the most effectiveness

**FIGURE 6:**
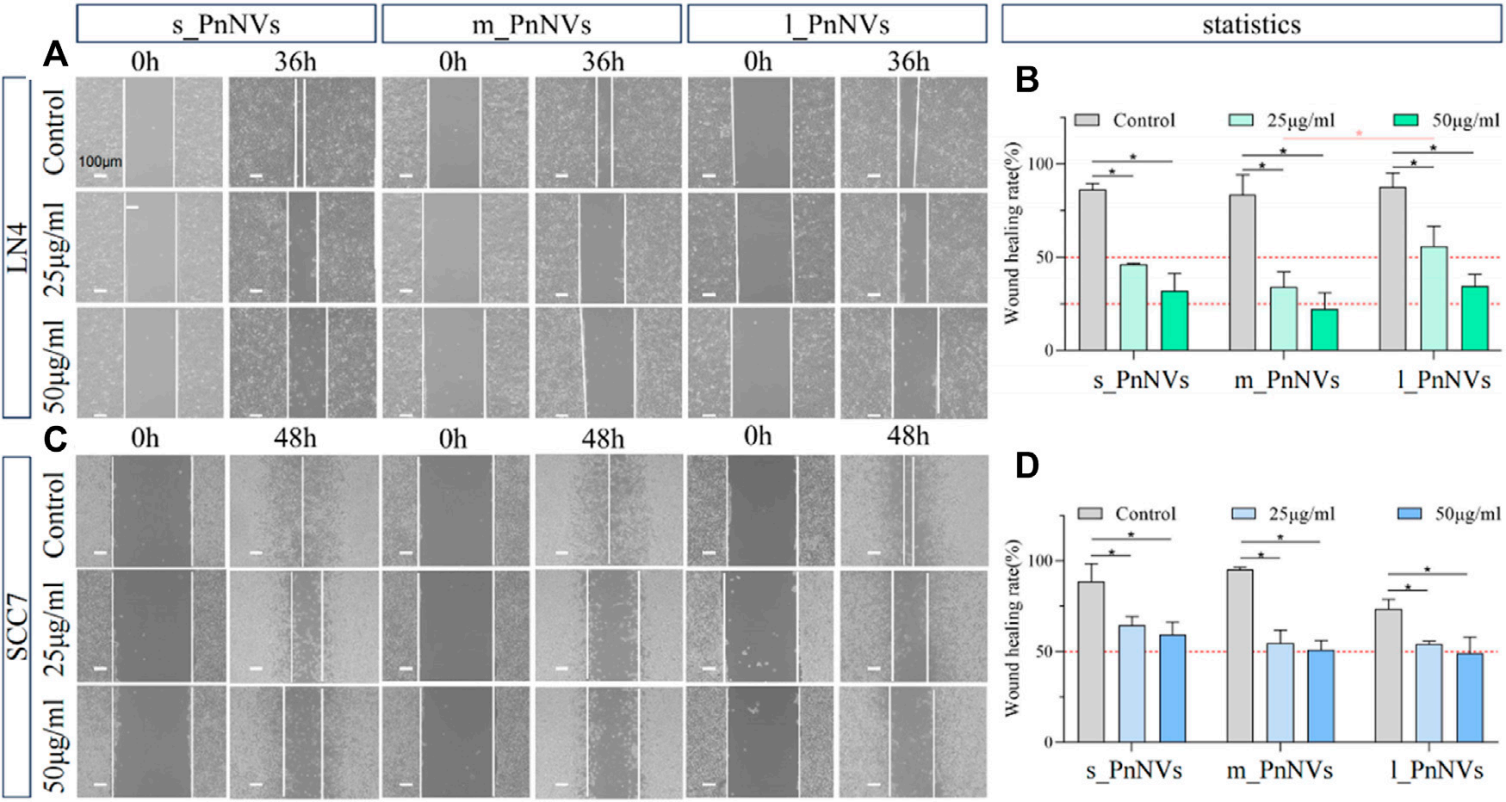
Effect of PnNVs in inhibiting cell migration activity. **(A)** Representative images of LN4 cell scratch healing at 0 h and 36 h after intervention with different concentrations of PnNVs. **(B)** Statistical analysis of the effect of LN4 cell scratch healing at 0 and 36 h after the intervention of different concentrations of PnNVs. **(C)** Representative images of scratch healing of SCC7 cells at 0 h and 48 h after intervention with different concentrations of PnNVs. **(D)** Statistical analysis of the scratch healing effect of SCC7 cells at 0 and 48 h after the intervention of different concentrations of PnNVs.

Metastasis and tumor recurrence are significant contributors to mortality in cancer patients, emphasizing the need for identifying drugs that can impede tumor cell motility (Fares et al., 2020). In this study, we assessed the efficacy of PnNVs in inhibiting the migration of squamous cell carcinoma cells through a scratch assay. Our results demonstrate that PnNVs effectively hindered the scratch healing ability of LN4 and SCC7 cells (Figure 6). Furthermore, statistical analyses revealed that m_PnNVs outperformed l_PnNVs in inhibiting cell migration at concentrations of 25 μg/mL and 50 μg/mL (Figures 6A, B). However, when conducting individual comparisons between SCC7 cells, no statistically significant differences were observed (Figures 6C, D). These findings indicatethat PnNVs possess the capability to hinder cell migration, with m_PnNVs exhibiting superior outcomes in certain instances.

### 3.5 Compositional analysis of PnNVs

**FIGURE 7:**
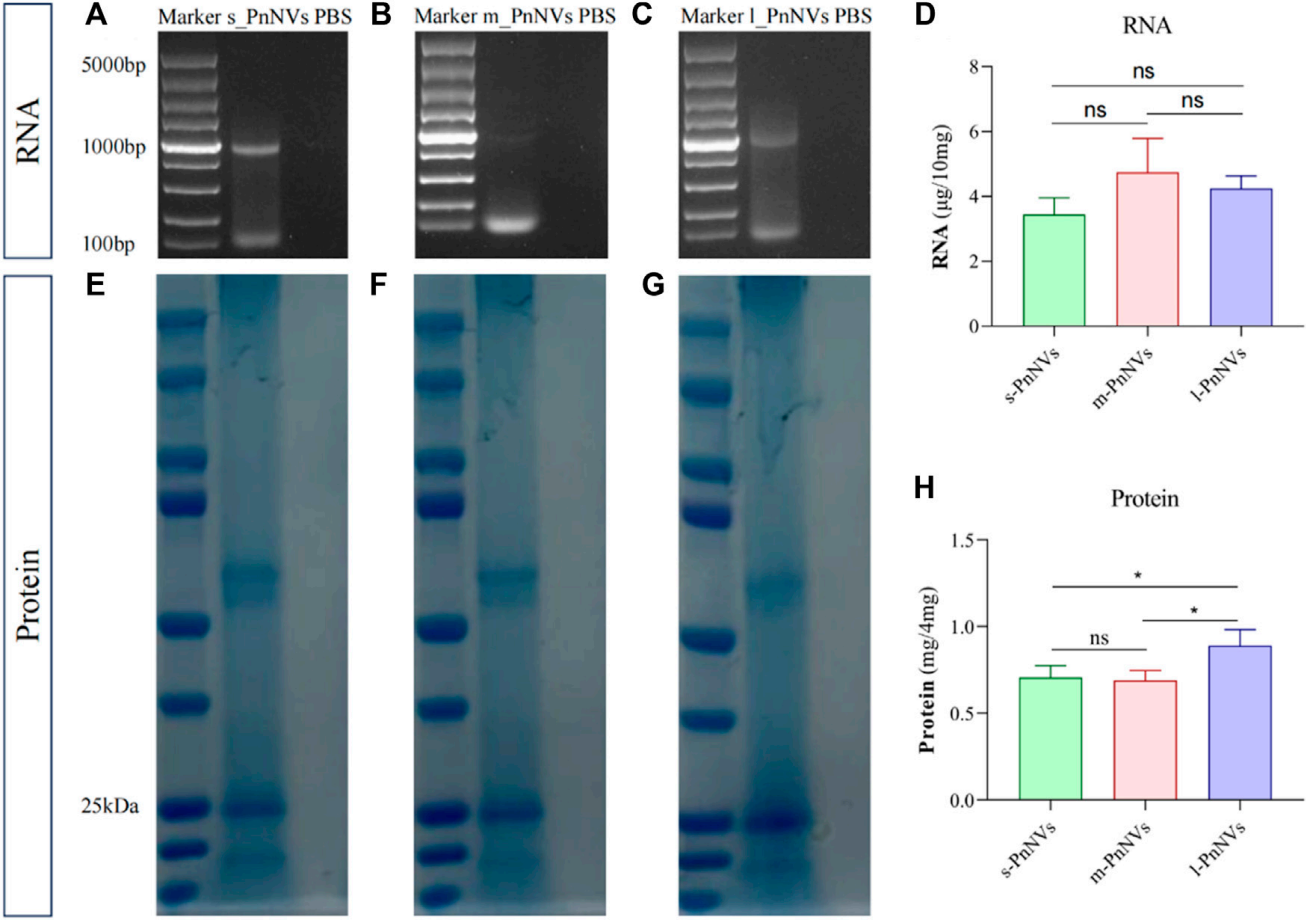
The protein and RNA in PnNVs. **(A)** Identification plot of RNA isolated from s_PnNVs **(B)** m_PnNVs **(C)** l_PnNVs. **(D)** Amount of RNA isolated in each 10 mg of PnNVs. **(E)** Identification plots of proteins isolated from s_PnNVs **(F)** m_PnNVs **(G)** l_PnNVs. **(H)** Amount of protein isolated in each 4 mg of PnNVs.

Numerous studies have been conducted on the metabolites of *Panax notoginseng,* with several components demonstrating promising anti-tumor properties (Tan et al., 2021). Additionally, our preliminarily analysis identified and quantified RNAs and proteins within these nanovesicles. Our findings indicate that PnNVs contain RNA (Figures 7A–C), although the content did not show significantly variation (Figure 7D). Furthermore, all PnNVs were around to contain proteins (Figures 7E–G), with a higher protein yield observed in nanovesicles of comparable quality from l_PnNVs (Figure 7H).

**FIGURE 8:**
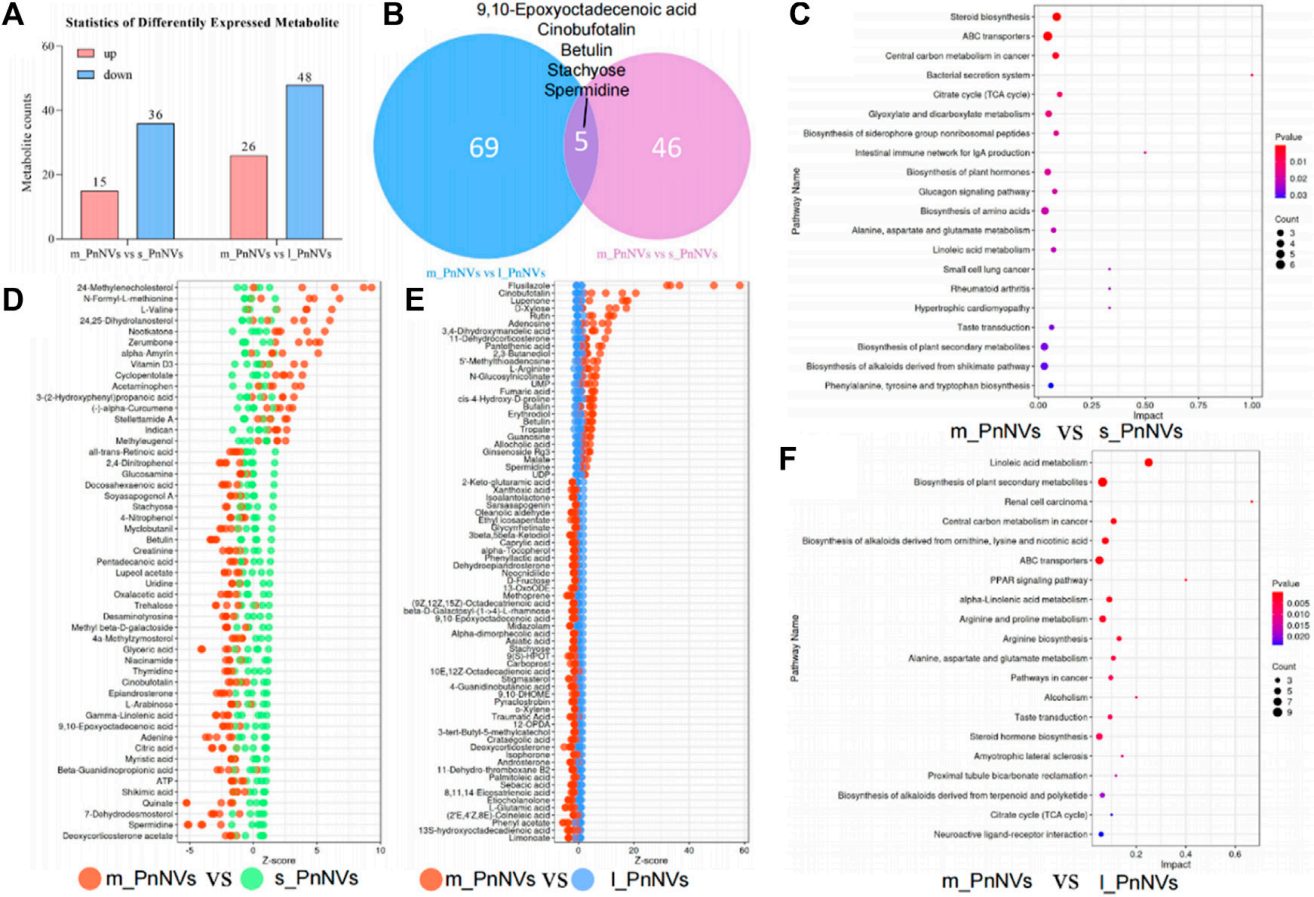
The metabolite analysis of PnNVs. **(A)** Statistical plots of metabolites with high and low expression compared to m_PnNVs, s_PnNVs and l_PnNVs. **(B)** Venn diagram of metabolites with differential expression compared to m_PnNVs, s_PnNVs and l_PnNVs. **(C)** KEGG enrichment analysis of differentially expressed metabolites in s_PnNVs and m_PnNVs. **(D)** Highly expressed metabolites in m_PnNVs compared to s_PnNVs. **(E)** Metabolites highly expressed in l_PnNVs compared to s_PnNVs. **(F)** KEGG enrichment analysis of differential metabolites in l_PnNVs and m_PnNVs.

The LC-MS analysis of PnNVs metabolites, revealed significant alterations in 52 metabolites in m_PnNVs compared to s_PnNVs, with 15 were upregulated and 36 downregulated metabolites (Figures 8A, D). Conversely, when comparing m_PnNVs to l_PnNVs, 74 metabolites exhibited changes, with 26 upregulated and 48 downregulated metabolites (Figures 8A, E). Among these altered metabolites, 9,10-Epoxyoctadecenoic acid, Cinobufotalin, Betulin, Stachyose, and Spermidine were identified as common changes, albeit with varying trends (Figure 8B). The metabolite expression levels were found to be lower in m_PnNVs compared to s_PnNVs, with these differentially expressed genes showing partial enrichment in tumor-related pathways (Figure 8C). Specifically, only 9,10-Epoxyoctadecenoic acid and Stachyose exhibited lower expression levels in m_PnNVs compared to l_PnNVs, while the remaining metabolites displayed higher expression levels. Cinobufotalin (Liu et al., 2019), Betulin (Amiri et al., 2020) and Spermidine (Madeo et al., 2018) are metabolites that have been found to be expressed at elevated levels and possess reported antitumor properties. Additionally, compounds like Rutin (Farha et al., 2022), which is also linked to antitumor effects, were detected in high concentrations. Analysis using the KyotoEncyclopedia of Genes and Genomes (KEGG) indicated that the identified metabolites are associated with cancer, including central carbon metabolism in cancer, pathways in cancer, renal cell carcinoma, small cell lung cancer. Furthermore, the analysis indicated potential regulation of numerous metabolism-related pathways, such as steroid biosynthesis, TCA cycle, biosynthesis of amino acids, and arginine biosynthesis. Further investigation is warranted to determine whether these regulatory mechanisms contribute to the observed inhibitory effects on tumor cell proliferation and migration. Further investigation is needed (Figures 8C, F). Nevertheless, the precise mechanisms underlying the variations in bioactivity warrant further investigation.

## 4 Discussion

PDNVs are tiny lipid vesicles found in plant cells that are believed to play crucial roles in signaling and substance transport processes within plants (Chen et al., 2023b; Kim and Kim, 2022). There is substantial evidence suggesting that the components present PDNVs possess potential pharmacological activities. For example, studies have shown that messenger RNAs (mRNAs) and small RNAs (sRNAs) contained in PDNVs can regulate the plant immune system and bolster their resistance against pathogens (Cai et al., 2018; Wang et al., 2024). Furthermore, secondary metabolites present PDNVs have been shown to exhibit pharmacological properties, including antioxidant, anti-inflammatory and anti-tumor affects, which could potentially benefit human health (Xu et al., 2023b). The formation and loading of nanovesicles compositions may be influenced by various factors such as gene expression, growth factors, and environmental conditions. For example, the components encapsulated within nanovesicles may vary depending on the size of the plant tubers, leading to variations in their pharmacological activities. Nevertheless, the mechanisms and patterns governing the sorting of substances within nanovesicles remain poorly understood (Woith et al., 2021; Viršilė et al., 2022).

*Panax notoginseng,* a significant medicinal plant in traditional Chinese medicine (Zhang et al., 2016), is utilized for its therapeutic properties in treating cardiovascular diseases due to its anticoagulant, antihypertensive and vasodilatory attributes. Additionally, *Panax notoginseng* exhibits promising anti-tumor capabilities owing to its antioxidant, anti-inflammatory, and immunomodulatory characteristics (Yang et al., 2018; Xie and Wang, 2023). The primary bioactive constituents of *Panax notoginseng* encompass saponin compounds, polysaccharides, flavonoids and phenolic acids (Liu et al., 2020). The variability of components in *Panax notoginseng* tubers of different sizes can impact their pharmacological activity. Research indicates that various parts of *Panax notoginseng* and its growth stages contain distinct types and quantities of active compounds (Dong et al., 2003). Specifically, saponins are predominant in early growth stages, while polysaccharides and flavonoids are abundant in mature stages. Consequently, variations in pharmacological effects may be observed in *Panax notoginseng* of different sizes. The tubers of *Panax notoginseng* are important source of PnNVs and have been identified as containing a high concentration of active ingredients within these nanovesicles. The active constituents present in PnNVs may play a crucial role in the pharmacological effects of *Panax notoginseng*.

Previous research has demonstrated the potential of PnNVs to mitigate ischemia-reperfusion injury by modulating the polarization state of microglial cells (Li et al., 2023). However, there is a lack of research on the anti-tumor properties of PnNVs. The variability in size of *Panax notoginseng*, a perennial plant, may result in the production of diverse secondary metabolites. The study aims to explore variations in the physical characteristics of nanovesicles produced by *Panax notoginseng* tubers of varying sizes, given their status as a tuberous plant. An analysis of these differences is crucial for understanding the relationship between nanovesicle properties and their source. Furthermore, an evaluation of variances in intracellular uptake efficiency and bioactivity of these nanovesicles could provide valuable insights into their functional mechanisms.

Differences in uptake efficiency of nanovesicles derived from various sources of *Panax notoginseng* were observed in our data, potentially attributed to variations in surface charge and composition of the nanovesicles. The surface charge of nanovesicles plays a crucial role in their interaction with the cell membranes, influencing cellular uptake (Fröhlich, 2012). Our findings indicate that s_PnNVs exhibited the lowest potential but the highest efficiency in cellular uptake, whereas those m_PnNVs displayed the highest potential but lowest efficiency in cellular uptake. Additionally, as the presence of proteins and lipids within nanovesicles may influence their intracellular uptake by interacting with cell surface receptors or other cellular mechanisms (Xu et al., 2022; Zu et al., 2021; Chen et al., 2024). This research has not fully characterized the proteins and other constituents of PnNVs, and further investigation into their components and co-localization analyses is anticipated to elucidate the variations in uptake efficiencies.

Furthermore, metabolomics data indicates a notable abundance of metabolites with strong anticancer properties in m_PnNVs compared to s_PnNVs. For example, Nootkatone has been shown to have antiproliferative effects on rectal cancer cells (Yoo et al., 2020), disrupting glucose metabolism, inhibiting the stemness of human breast cancer stem cells (Nguyen et al., 2022), and reducing the sensitivity of tumor cells to certain drugs (Hung et al., 2019). Additionally, Zerumbone (Prasannan et al., 2012), and Methyleugenol (Groh and Esselen, 2017) have been identified as having anti-tumor activity. Importantly, m_PnNVs exhibit a higher expression of these metabolites with anticancer properties compared to l_PnNVs. Metabolites such as guanosine (Zhao et al., 2020), rutin (Ghanbari-Movahed et al., 2022), spermidine (Al-Habsi et al., 2022), and cinobufotalin (Han et al., 2021) have demonstrated significant antitumor effects. The metabolites identified in this study exhibited high expression in m_PnNVs, with Ginsenoside Rg3 (Sun et al., 2022; Zhu et al., 2023), a potent antitumor compound found in *Panax notoginseng*, showing particularly elevated expression. Furthermore, these metabolites were also identified in the KEGG enrichment analysis of pathways related to tumor development. The differential enrichment of these highly expressed metabolites may play a role in the improved effectiveness of m_PnNVs, although further research is needed to elucidate the exact mechanisms involved.

It is important to acknowledge that the observed disparity in the size of *panax notoginseng* or ginseng may be attributed to various factors such as the duration of planting, preservation conditions, timing of harvest, as well as levels of dryness and rainfall during the planting phase (Liu et al., 2021b; Tang et al., 2024). This complexity presents a challenge to the study’s quality control and necessitates further investigation by experts in the field of agriculture. During the growth of plants, particularly non-perennial species like Artemisia annua and dandelions (Thu et al., 2011), the potential variations in their nanovesicles as they progress through germination and flowering in response to seasonal changes warrant further investigation. Additionally, researchers studying herbaceous plants in this area are also concerned with the batch stability of PDNVs.

Despite the initial evaluation of nanovesicles derived from *Panax notoginseng*, it is important to recognize that certain aspects have not been thoroughly examined in the study. One primary limitation is the absence of data derived from animal experiments to support the findings. While existing research has concentrated on squamous cell carcinoma cell models, the response of various cancer cell types or normal cells to PnNVs remains inadequately explored. To address this research gap, future studies could incorporate a diverse range of cell models to investigate the therapeutic efficacy of nanovesicles across different cell types. Such investigations may elucidate the widespread utility and therapeutic potential of nanovesicles, offering a more foundation of data. Moreover, there is a lack of comprehensive research on the distribution and metabolic kinetics of nanovesicles *in vivo*. Despite the potential of nanovesicles for drug delivery, further detailed investigations are required to elucidate their *in vivo* behavior. This necessitates examinations of the stability of nanovesicles within the circulatory system, their distribution in tissues, and their metabolic pathways. Enhanced knowledge of the *in vivo* behavior of nanovesicles is crucial for maximizing their efficacy in drug delivery and therapeutics applications, and for refining their utilization.

## 5 Conclusion

In summary, the objective of this research was to extract nanovesicles from *Panax notoginseng* tubers of varying sizes and analyze the effects of tuber size on nanovesicles composition, cellular uptake efficiency, and bioactivity. The results indicated noticeable differences in the characteristics of the PnNVs. Notably, s_PnNVs demonstrated, lowest zeta potential, and the highest cellular uptake efficiency after 24 h. Conversely, m_PnNVs exhibited the smallest size but the lowest cellular uptake efficiency after 24 h. Notably, PnNVs exhibited pronounced inhibitory effects on the proliferation and migration of LN4 and SCC7 cells, with m_PnNVs displaying the greatest efficacy. Untargeted metabolomics analysis unveiled a varied compound profile within PnNVs, with metabolites abundantly expressed in m_PnNVs being enriched in anti-tumor pathways. Subsequent compositional analysis identified a protein and RNA composition in PnNVs, particularly in l_PnNVs which contained a higher protein content. In conclusion, our research has presented initial findings indicating that PnNVs derived from varying sizes of *Panax notoginseng* tubers exhibit unique physical characteristics, compositional variances, and effects on cellular activity. These results establish a fundamental basis for further exploration of the therapeutic potential of PnNVs as anti-tumor agents. Nevertheless, further research is warranted to elucidate the biological properties, mechanisms of action, and potential clinical utility of PnNVs.

## Data Availability

The raw data supporting the conclusions of this article will be made available by the authors, without undue reservation.
